# Burden of Fungal Infections in Colombia

**DOI:** 10.3390/jof4020041

**Published:** 2018-03-21

**Authors:** Carlos Arturo Alvarez-Moreno, Jorge Alberto Cortes, David W. Denning

**Affiliations:** 1 Internal Medicine Department, School of Medicine, Universidad Nacional de Colombia, Bogotá 111321, Colombia; caalvarezmo@unal.edu.co; 2 Clínica Universitaria Colombia, Bogota 111321, Colombia; 3 The University of Manchester and National Aspergillosis Centre, Wythenshawe Hospital Manchester, Manchester M13 9PL, UK; ddenning@manchester.ac.uk

**Keywords:** mycoses, invasive fungal mycoses, lung diseases fungal, candidiasis, aspergillosis, invasive fungal infections, dermatomycoses, Colombia

## Abstract

Data with respect to the epidemiological situation of fungal diseases in Colombia is scarce. Thus, the aim of this study is to estimate the burden of fungal infections. A population projection for 2017 from the Colombian Department for National Statistics was used, as well as official information from the Ministry of Health and National Institute of Health. A bibliographical search for Colombian data on mycotic diseases and population at risk (chronic obstructive pulmonary disease, HIV infection/AIDS, cancer, and transplant patients) was done. The Colombian population for 2017 was estimated at 49,291,609 inhabitants, and the estimated number of fungal infections for Colombia in 2017 was between 753,523 and 757,928, with nearly 600,000 cases of candidiasis, 130,000 cases of aspergillosis, and 16,000 cases of opportunistic infection in HIV, affecting around 1.5% of the population. In conclusion, fungal infections represent an important burden of disease for the Colombian population. Different clinical, epidemiological, and developmental scenarios can be observed in which fungal infections occur in Colombia.

## 1. Introduction

Access to data on the burden of disease is critical for public health actions. Globally, fungal infectious diseases of the lung have been estimated to affect tens of millions and lead to over a million deaths annually [1]. The total number of fungal infections affecting the human race is still unknown, especially in poorly developed countries. Identification of the burden of disease aids in establishing public health measures as well as in determining the diagnostic and therapeutic needs of the population.

Colombia is situated in the north part of South America. It has a unique environment, with part of the Amazon basin situated in the south part of the country. The Andes mountains cross the country alongside two big rivers (Magdalena and Cauca), creating almost every type of weather niche, from deserts in the north part, to jungle in the Amazon basin and the Pacific coast, and even snow peaks, together with extensive areas of coffee and other plantations. In this scenario, large cities with corresponding pollution problems have developed (for example Bogota, 2600 m above sea level with an estimated population of 8 million) with severe inequities between the rural and urban populations. There are certain fungal infections related to advanced healthcare (intensive care units, transplant procedures, immunosuppression) and an important but difficult to define burden of endemic mycoses.

The country has a long tradition of research on endemic fungal infections, such as paracoccidiodomycosis, but a figure on the annual incidence and prevalence fungal diseases is lacking. It is reported that Colombia comes second to Brazil and Venezuela in terms of number of cases of paracoccidioidomycosis [2]. Histoplasmosis is endemic in the western areas of the country, while it is endemic in Central America and some areas of Brazil [3]. There is also information showing a higher incidence of candidemia in Colombia in relation to Brazil and other Latin American countries [4]. *Cryptococcus* infections are endemic among patients with a similar serotype of AIDS in the region [5]. The objective of this study is thus to estimate the burden of serious fungal diseases in Colombia.

## 2. Materials and Methods

### 2.1. Literature and Data Search

The burden of serious fungal infections in the country was estimated using the methodology established by Leading International Fungal Education (LIFE), which has been used previously for different countries in the world [6]. A search for literature about fungal infections in Colombia was done to identify the key epidemiology papers in English or Spanish, as well as grey literature and official reports by governmental offices. The electronic database Medline, LILACS (Literatura Latinoamericana de Información en Ciencias de la Salud), Google, Google Scholar, and Embase were used with the terms “*Cryptococcus*”, “*Pneumocystis*”, “*Aspergillus*”, “*Candida*”, “*Mucor*”, “*Histoplasma*”. “*Sporothrix*”, “fungal keratitis”, “tinea”, “lobomycoses”, “*Paracoccidiodes*”, and “Colombia”.

### 2.2. Frequency Estimates

Incidence rates were estimated per 100,000 habitants, taking into account the projection of the Colombian Census. Prevalence was calculated for the chronic forms of aspergillosis and the cases of tinea.

### 2.3. Data Estimation Sources

We estimated the burden of serious fungal infections based on the specific data available for the country, as well as extrapolation of data on people at risk in the country. Colombia demographics for 2017 were obtained from the National Department for Statistics (Departamento Administrativo Nacional de Estadística (DANE) as per its acronym in Spanish), and the official projections on population based on the last census (carried out in 2005).

The number of cases of HIV infection was obtained from national data, itself based on insurance claims and HIV programs from around the country [7], since reporting is mandatory for cases of HIV infection. For fungal infections among AIDS patients, new patients with AIDS were considered at risk of these fungal infections [7], and 2000 cases were added to the risk population to compensate for missing data. Cases of *Pneumocystis* infection were calculated based on international data on the incidence of these fungal infection among AIDS patients [8,9].

Candidemia cases were calculated by estimating the number of cases in intensive care unit based on the observed epidemiology in patients hospitalized in such wards in Colombia [10,11], assuming a mean stay in the ICU of five days and an occupancy of 80%. For Candida peritonitis, we assumed that the rate was the half of the ICU candidemia rate [12]. To establish the prevalence of recurrent vulvovaginal candidiasis (VVC), an estimate of 5% of women between the ages of 18 and 50 years (for the 2017 national projection) was used [13]. To estimate the number of cases of oral candidiasis, we assumed that 90% of untreated HIV patients presenting with AIDS [9] would have this condition, and for the number of esophageal cases, we assumed 20% of new AIDS cases and 0.5% of those on treatment with anti-retroviral therapy [14].

To estimate the impact of aspergillosis, we calculated the number of cases of chronic pulmonary aspergillosis (CPA) after pulmonary TB according to methodology previously described [15,16]. Briefly, we used the 2016 number of annual new pulmonary TB cases [17]. The number of cases of CPA in cavities was calculated by applying 12% as the proportion of patients that developed cavities, and then calculating 22% of this figure, which represents the incidence of CPA in cavities. In addition, we included a proportion of 2% of TB patients that did not develop cavities (78%) as having CPA. Then, we estimated a five-year period of prevalence of CPA, assuming a 15% annual mortality or surgical cure rate. Finally, assuming TB is the underlying diagnosis in 33% of cases, we estimated the total number of CPA cases [18]. In addition, the number of invasive aspergillosis (IA) cases was considered for different populations at risk (10% of patients with acute myeloid leukemia; 0.5%, 4%, and 6% of renal, lung, and heart solid transplant patients, respectively). The annual number of transplant recipients was taken from official data [19] and the number of acute leukemia patients was estimated from reports of the National Cancer Institute in Colombia, which reported an incidence of 2.21 cases per 100,000 persons [20,21].

The number of chronic obstructive pulmonary disease (COPD) cases was estimated using the figure of 8.9% in terms of prevalence among the Colombian population aged 40 years or more [22], the proportion admitted to hospital each year (20.3% after United Arab Emirates), and considering that IA occurred in 1.3% of these patients [23]. Finally, the number of IA cases among lung cancer patients was estimated from data extrapolated from a large dataset from China (2.6%) [21,24]. For our analysis of adult asthmatics, we used an asthma rate of 6.5% [25]. Estimation of allergic bronchopulmonary aspergillosis (ABPA) prevalence assumes that 2.5% of adult asthmatics develop this complication, and 15% of adults with cystic fibrosis [26,27], while estimation of severe asthma with fungal sensitization (SAFS) prevalence assumes that 33% of the worst-affected 10% of adult asthmatics or 3.3% of the total number of adults with asthma are affected [28].

Cases of cryptococcal infections were estimated using HIV information and data published from national surveys on cryptococcal infection in the country [29,30]. According to a recent global estimate, cryptococcal antigenemia is found in 6% of patients with a CD4 count of less than 100 cells per µL [31]. According to Colombian national surveys, between 78.1% and 83.5% of the cases were related to HIV infection [29,30]. Histoplasmosis cases were estimated using the national data on HIV and a published Colombian survey [32]. The relative frequency of histoplasmosis among HIV-infected patients in relation to the cryptococcosis infection was 3:1 (that is 1 case of histoplasmosis per every 3 cases of cryptococosis). According to the national survey, 80% of the reported cases occurred in HIV-infected patients [32]. Among patients with HIV and lymphadenopathy in endemic areas, the ratio of tuberculosis to histoplasmosis was 4:1 [33]. The number of cases of immunosuppression was estimated from a Colombian survey [32]. Mortality for opportunistic fungal infections was calculated using Colombian data for cryptococcosis and histoplasmosis [34,35] and international figures for *Pneumocystis* pneumonia [36].

Sporotrichosis cases were estimated according to the relative frequency found in a reference center with data from some areas of the country [37]. Paracoccidiodomycosis cases were estimated using a study defining the observed incidence over a 50-year period up to 1999 [38].

Cases of fungal keratitis were calculated using a study on infectious keratitis in a reference center covering a population of nearly 2 million inhabitants [39]. In this case series, 5% of the cases were fungal. The incidence in that population was eight cases per year. *Tinea capitis* cases were seen in children adopted from Colombia [40] and no Colombian publication was found on the issue. The population with the lowest socioeconomic level according to the national statistics was used to estimate the number of cases in children aged from 1 to 5 years old.

An estimate of the number of cases of rare fungal infections such as mucormycosis and lobomycosis, both using reported cases in the country [41,42,43], was done based on the population at risk. Although older descriptions of chromoblastomycosis or mycetoma could be found, there is no recent data on these conditions.

## 3. Results

### 3.1. Population and Risk Factors

Colombia is situated in the north coast of South America. With coasts over the Caribbean (Atlantic) and the Pacific oceans, the country contains various climate environments including the jungle of the Amazon basin, jungle that extends from the Pacific rim up to the Atlantic coast, mountains and valleys from the Andean mountain system (that extends from Argentina and Chile to Venezuela), and even deserts in the north and extended plains in the east. Over 70% of the population inhabits the crowded big cities. The last population census in the country was performed in 2005 and we used the 2017 projections for our estimates. Table 1 shows the demographic data from the country as well as the estimated numbers for population and patients at risk for several pathologies or interventions. Children (under 18 years old) correspond to 31.3% of the population.

In Colombia, HIV-infected patients have access to antiretroviral treatments supplied by insurers (public or private). HIV infection is subject to mandatory reporting, and the costs of medical care are covered by a public account (Account for High Cost—*Cuenta de Alto Costo* in Spanish) that also includes chronic renal failure patients with dialysis. In the last report [7], for 2016, there were 73,465 known patients infected with HIV in the country, but only 65,044 of them were retained in the health care system and receiving antiretroviral treatment. These gaps in the health care system may be aggravated by major underreporting of HIV diagnosis. The estimated country prevalence for HIV as reported by insurers is 0.15% [7], while the figure obtained by UNAIDS is 0.5% which allows us to conclude that close to 30% of people are unaware of their HIV-positive status. Women represent 25.9% of the HIV population [7]. The main form of HIV transmission is through sexual intercourse, especially among men having sex with men. Overall, 75% of the HIV population is concentrated in the cities, while the prevalence is lower in rural areas, eastern plains near the Venezuelan border, and in the Amazon basin. In 2016, 8209 new cases of HIV were reported, and 34% of the newly diagnosed patients had a CD4 count of less than 200 cells/µL or AIDS-defining opportunistic illnesses, while only 25.1% had a CD4 count over 500 cells/µL. Using the above data as a basis, we calculated the risk of developing an opportunistic infection due to a low CD4 count at the time of diagnosis (AIDS) from the new cases (36%), and added to that figure the possible cases due to lack of healthcare.

Other risk factors evaluated in the Colombian population included the incidence of tuberculosis [17] as reported in the health care system, COPD [22], asthma [25], and solid organ or hematological transplants. Estimates from the Colombian Institute of Health suggest a donation rate between 20 and 30 cases per 1,000,000 inhabitants per year.

Table 2 shows the estimated number of cases for the different fungal infections, categorized by risk groups, while Table 3 shows the comparison with the global incidence.

### 3.2. Candidiasis

The number of ICU beds in the country has been estimated at around 2310 [44]. In Colombia, the overall incidence of candidemia in the ICU was reported to be 1.4% [10]. Incidence was estimated in two studies as 2.3 cases per 1000 ICU-days and 1.96 cases per 1000 hospital admissions [4,10]. A significant trend towards an increased number of cases of candidemia has been noted [10]. There has been also a shift in the number or cases caused by *Candida albicans* in contrast to non-*C. albicans* cases, with more recent studies showing an increase in the cases of *C. tropicalis*, *C. parapsillopsis*, and *C. glabrata* [4,45,46,47]. A low frequency of fluconazole resistance has been observed among the three main identified species [4].

### 3.3. Aspergillosis

The estimated number of cases of different forms of aspergillosis is probably about 130,000, with a high burden of disease in patients with respiratory diseases due to the incidence of both asthma and COPD in Colombia, only exceeded by the population affected by vulvovaginal candidiasis (Table 2). The presence of chronic pulmonary aspergillosis post TB is also estimated in about 2000 people, with an annual incidence after TB of 458 cases (1/100,000). Asthma in adults is relatively common, with over 2 million affected, so both ABPA and SAFS prevalence figures are high at 106 and 140 per 100,000, respectively. These estimates lack confirmatory data from Latin America of ABPA prevalence and need validation with local studies. Finally, the number of cases of invasive aspergillosis calculated was 2820, of which 361 cases were related to recipients of organ transplantation, especially stem cell transplantations.

### 3.4. Opportunistic Infections Related to HIV and Other Conditions Involving Immunosuppression

Solid organ and bone marrow transplants are recorded in a central register by the Colombian National Institute of Health [48]. The annual number of renal transplants has been stable, while the number of transplants of other solid organs has slightly increased. There is no formal data on the number of cases of lupus or on the frequency of the use of immunosuppressant drugs, but information on the use of all kind of these medications, as well as tumor necrosis factor inhibitors, is available in the Colombian health market. The numbers of patients with cancer are registered centrally by the National Institute of Cancer. Some data on the number of new cases of leukemia and other tumors are available for some years [20,21]. The number of annual cases of systemic fungal opportunistic infections in HIV patients in Colombia (histoplasmosis, cryptococcosis, and pneumocystis cases) was estimated at around 2468, with 398 deaths (16%).

### 3.5. Endemic Mycoses and Other Fungal Infections

**Figure 1:**
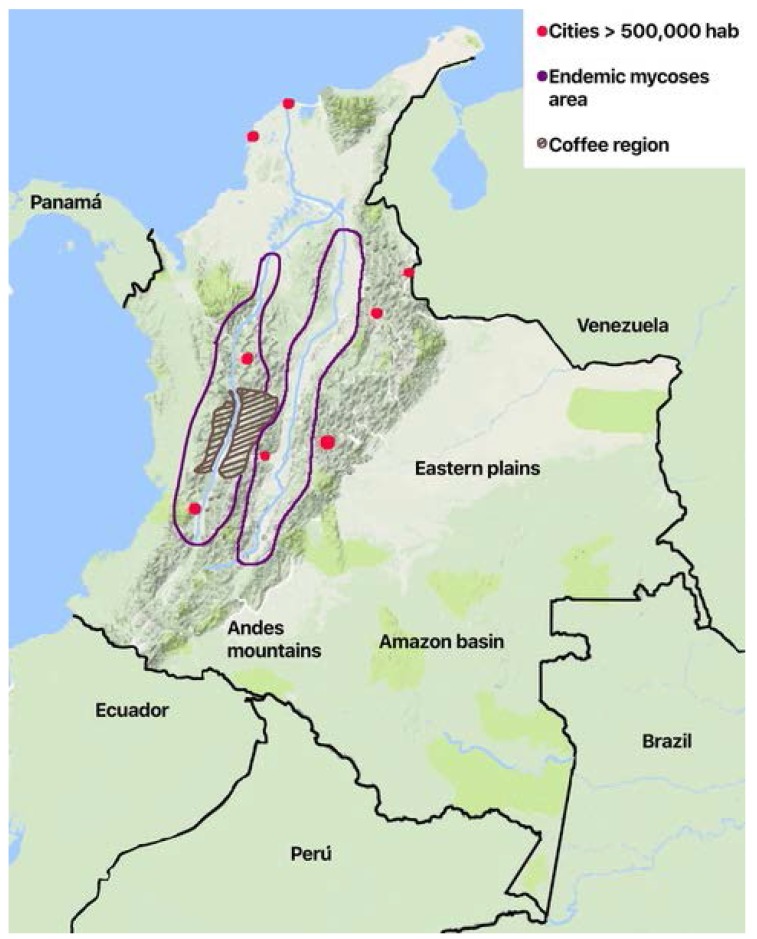
Schematic map of Colombia showing geographical and ecological areas for endemic mycoses.

The geographic distribution of the cases varies according to the risk factors and the ecoepidemiological conditions for the different diseases. Histoplasmosis and paraccocidiodomycosis have a regional distribution. Histoplasmosis is confined to the two main river valleys across the Andean divisions in the country (Figure 1): The Magdalena River and the Cauca River. Paracoccidiodomycosis has been localized to the coffee region in the central area. While the rural population has diminished because of migration, economic development of the cities, and the civil war that took over 50 years in the country (1960–2017), it is now affected by formerly uncommon diseases like mycetoma. There are local reports of sprotrichosis, fungal keratitis, and lobomycoses [37,39,41], the latter in indigenous Amerindian population in the plains in the eastern part of the country on the border of Venezuela and the Amazon jungle.

## 4. Discussion

This is the first systematic approach to estimating the number of common and serious fungal infections in the country. It is based on a proposed methodology that has been widely used, including in neighboring countries [49,50,51]. This is a challenging task due to the lack of a mandatory surveillance system for the majority of fungal diseases. While some official information is present for some risk groups, such as HIV and transplant patients, there is a general lack of data about some uncomplicated fungal infections, and also about some that are more serious or even lethal.

In Colombia, there are certain scenarios with respect to fungal infections, which differ in comparison to more developed countries. One is related to healthcare, as there is an increased number of patients with chronic diseases involving immunosuppression (because of the disease itself or because of the immunosuppressant medications used). The majority of cases of systemic candidiasis in transplanted patients form a part of this scenario. Significant efforts have been made to develop the health care system, allowing the population to access critical care units, high complexity hospitals, new and expensive medications, and transplant procedures, etc., with an ever-increasing number of cases. In this context, a relatively higher frequency of candidemia among patients in the ICU has been noted, as compared with the frequency seen in more developed hospitals [4,52]. Most likely, the lack of adequate hygiene control measures in hospitals, which is reflected in a higher frequency of hospital-acquired infections, might explain this phenomenon [53]. In addition, a new challenge has arisen with the appearance of *Candida auris*, a multidrug-resistant pathogen which is a notorious healthcare-associated yeast causing invasive infections with high rates of clinical treatment failure, which has already been described in hospitals in Colombia [54].

Another important scenario in the country is the situation of endemic mycoses. While it is commonly referred to in infectious disease and travel medicine textbooks, the number of cases and the risk factors have changed. Histoplasmosis is a good example of this situation. Previously related to agricultural activities and speleology, nowadays more than 70% of the patients have HIV infection as a comorbidity [32,55]. Old studies with histoplasmin defined the geographical areas where the infection might be found around the main two rivers of the country [56] and calculated a population at risk of near 6 million inhabitants 40 years ago (Figure 1). Although the endemic areas have probably not changed, the main risk factor is HIV infection and the rural population has clearly declined. Paraccocidioidomycosis has steadily declined in incidence. While serologic studies identified similar areas at risk [38], the lack of association with HIV has not changed the clinical scenarios where the disease is identified. Two challenges may appear, affecting the incidence of these diseases in the near future. First, there may be an increase in cases related to reactivations resulting from the greater number of immunosuppressed patients due to the use of immunosuppressive drugs such as steroids, tumor necrosis factor inhibitors, and new biologic therapies [57]. Second, the armed conflict in Colombia has ceased, allowing the general population to access endemic geographical areas including the Amazon jungle, which will facilitate the appearance of new infections.

The other major clinical risk scenario is related to the incidence of HIV infection. Our estimates depend deeply on the quality of the mandatory reporting. The actual number of HIV cases might be underestimated, and the fact that the majority of cases appear in late clinical stages suggest that our numbers might be very conservative. Furthermore, in rural areas HIV infection continues to be stigmatized and therefore patients do not access the health system or do so late, increasing the risk of occurrence of mycotic opportunistic infections. Unfortunately, in the same areas diagnostic tools are limited, making it difficult to know with certainty the true burden of fungal diseases. It is necessary for the endemic areas to stimulate the use of diagnostic tests not only for endemic fungi but also opportunistic fungal infections in this population at risk. Our data suggest the need for earlier identification of HIV patients with implementation of measures to identify serious infections (like cryptococcosis, histoplasmosis and *Pneumocystis* pneumonia) which should result in the prevention of a substantial number of HIV-related deaths in the country [58].

In Colombia, there is only one publication that describes the presence of cases of APBA in patients with cystic fibrosis [59]. There is no local data addressing any potential role of *Aspergillus* spp. in COPD patients, despite the frequency of this disease (8.9%). Currently, we are evaluating the incidence of *Aspergillus fumigatus* during exacerbation episodes in chronic obstructive pulmonary disease patients from Colombia. In the same way, few studies have evaluated the role of *Aspergillus* causing allergic reactions in the Colombian population with asthma, despite its prevalence (6.9%). A study in Medellin with fungi sensitization in a prick test population under 70 years old found that a multisystemic pattern defined as the simultaneous coexistence of asthma, rhinitis, and dermatitis was associated with *Aspergillus fumigatus* sensitivity, with a risk ratio of 4.38 (95% CI: 1.12–17.2) [60]. Given the prevalence of COPD and asthma in the Colombian population, it is necessary to optimize early diagnosis and avoid complications of invasive presentations or pulmonary complications of SAFS or APBA. Finally, the estimations of both IA and CPA cases are greater than what is currently observed, probably related to the absence of a compulsory register of this disease, the absence of adequate diagnostic methods around the country (especially in areas with higher incidence of tuberculosis), and lack of clinical awareness, among other factors. The impacts of these invasive and chronic forms may worsen if one considers the recent description of azole-resistant *Aspergillus fumigatus* in the environment, which limits therapeutic options to intravenous therapy in most cases [61].

There is an important lack on information on common but insidious fungal skin and vaginal infections. No local reports on the incidence, prevalence, or burden of the problem have been found, despite the fact that according to worldwide epidemiology, these are the most frequently found fungal infections [62].

**Figure 2:**
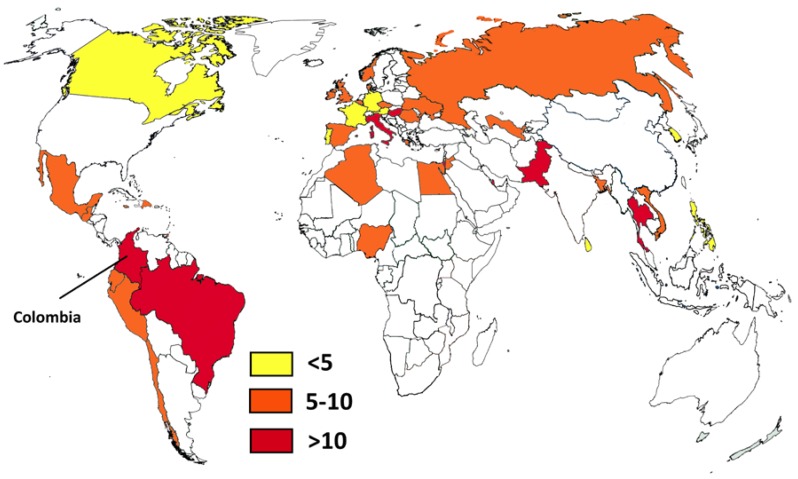
Country estimates of candidemia per 100,000 habitants. Data from references [62,63,64,65] and this study.

It is interesting to compare the Colombian situation with that of some neighboring countries like Ecuador and Peru [49,50]. For example, the number of HIV patients not receiving antiretroviral treatment in Peru is higher than in Colombia, while the number of cases in Ecuador is not known. While Peru has a smaller population, there are important differences in the access to health care. Colombia has a bigger health care system with better access. This is reflected in the number of solid organ transplants, which is eight times higher in Colombia. In the case of tuberculosis there is a similar situation: the number of cases in Peru is almost double that of Colombia, and Ecuador has half the number of notified cases (with a population one-third that of Colombia). In addition, Colombia, Peru, and Ecuador share the Andean mountains and the Amazon basin environment, which provide ecological areas for some endemic diseases. It is also interesting to compare local estimates on systemic candidiasis with worldwide figures (Figure 2) [62,63,64,65]. Brazil, another neighboring country, probably has the highest incidence (14.9/100,000), but Colombia is close behind (12.8/100,000). As mentioned, these high rates could be related to infection control problems in both countries. Pakistan is rated first in the incidence of *Candida* infections (21/100,000) and has also problems with *C. auris* [66] like Colombia. Other opportunistic fungal infections such as invasive aspergillosis are found in Colombia, with an incidence similar to that of other South American countries [62], and not too distinct from the incidence in Europe. This could be related to better access to cancer treatment, an increasing number of patients receiving immunosuppressant drugs, and the large number of patients with COPD.

There are several limitations to our estimates. The first (already mentioned) is the lack of information on several of the diseases mentioned here, especially those that are more prevalent, including recurrent vulvovaginal candidiasis and tinea capitis. Another important limitation is the lack of any mandatory reporting for serious fungal infections, such as cryptococcosis and histoplasmosis. Since several non-culture diagnostic tests are not widely available, the reported incidence or prevalence might be underestimated. This may also be the case for candidiasis, in which the standard diagnostic test (i.e., blood culture) has low sensitivity. Additionally, fungal infections may be underdiagnosed because of low clinical awareness, as may be the case with respect to the relationship between *Aspergillus* and asthma. As mentioned, there is little information on the subject in the country.

In conclusion, fungal infections represent an important burden of disease for the Colombian population. However, to better understand the impact of fungal diseases and therefore optimize their management, it is necessary to improve the diagnostic tools throughout the country and improve clinical awareness in special populations not only for early detection but also for accurate treatment.

## Figures and Tables

**Table 1:** Demographic and health data from Colombia (2013–2017) from groups of patients at risk for fungal infections. COPD: chronic obstructive pulmonary disease.

Population	Number
Total population, 2017	49,291,609
Total adults, 2017	33,843,324
Adult women, 2017	17,403,058
Women 18–50 years-old, 2017	11,832,865
HIV population	73,465
HIV population receiving ART	65,044
Pulmonary tuberculosis, 2016	10,442
Asthma in adults	2,090,506
COPD in adults over 40 years	921,260
COPD patients admitted to hospital	189,117
Lung cancer, 2011	3985
Acute leukemia, 2017	1089
Renal transplant recipients, 2014	732
Liver transplant recipients, 2014	211
Allogeneic stem cell transplant, recipients, 2014	172
Heart transplant recipients, 2014	72
Lung transplant recipients, 2014	10
Critical care beds, 2013	2310

ART: Anti-retroviral tereatment.

**Table 2:** Estimated burden of fungal infections.

Fungal Infection	None	HIV/AIDS	Respiratory Disease	Cancer and Immunodeficiency	Critical Care and Surgery	Total	Rate/100,000 Inhabitants
Candidemia				4407	1889	6296	12.8
Candida peritonitis					944	944	1.9
Oral candidiasis		9150				9150	18.6
Oesophageal candidiasis		4269		638		4907	10
Recurrent Candida vaginitis (>4×/year)	591,643					591,643	2401
Invasive aspergillosis				361	2459	2820	5.7
Chronic pulmonary aspergillosis post TB *			2106			2106	4.3
Chronic pulmonary aspergillosis—all			8426			8426	49
Allergic bronchopulmonary aspergillosis (ABPA)			52,268			52,268	106
Severe asthma with fungal sensitisation (SAFS)			68,987			68,987	140
Cryptococcal meningitis	65	719		54		838	1.7
*Pneumocystis* pneumonia		1525				1525	3.1
Mucormycosis				99		99	0.2
Histoplasmosis	39	225		22		286	0.6
Sporothricosis	55					55	0.1
Fungal keratitis	182					182	0.4
Tinea capitis	12,134					12,134	25
Lobomycoses	2					2	0.01
Paracoccidiodomycoses	246					246	0.5
Total fungal infection burden	604,366	15,888	129,681	5581	5292	760,808	1543

* Note that the total fungal infection burden does not include the number of estimated cases of chronic pulmonary aspergillosis after TB, because those cases were already accounted for in the total for all chronic pulmonary aspergillosis cases.

**Table 3:** Annual incidence of selected fungal infections in Colombia in comparison with the global incidence.

Fungal Infection	Colombian Incidence	Global Incidence	Rate/100,000
Histoplasma infection	286	500,000	0.6
Invasive candidiasis	6296	750,000	12.8
Invasive aspergillosis	2820	300,000	5.7
*P. jirovecii* pneumonia	1525	500,000	3.1
Cryptococcosis in AIDS	719	223,000	1.5
Mucormycosis	99	>10,000	0.2
